# Deciphering the significance of neutrophil to lymphocyte and monocyte to lymphocyte ratios in tuberculosis: A case-control study from southern India

**DOI:** 10.12688/f1000research.150685.2

**Published:** 2025-01-08

**Authors:** Poorva Bakshi, Rakshatha Nayak, Sharada Rai, Shikha Jayasheelan

**Affiliations:** 1 Department of Pathology, Kasturba Medical College, Mangalore, Manipal Academy of Higher Education, Manipal, Karnataka, 576104, India

**Keywords:** NLR, MLR, Tuberculosis, Infectious diseases, Hematology

## Abstract

**Background:**

Diagnosis of tuberculosis (TB) in resource-limited countries relies primarily on bacteriological confirmation using Ziehl-Neelsen (ZN) stain on sputum or other representative samples. However, this method has low sensitivity due to suboptimal sampling and techniques. While AFB culture remains a gold standard for diagnosing TB and other mycobacterial infections, its limitations include slow turnaround time, and the requirement for specialized resources and expertise. Neutrophils, monocytes, and lymphocytes are crucial in the pathogenesis of granulomatous inflammation and immune reactions. We investigated the usefulness of the haematological parameters and their ratios, like the Neutrophil to Lymphocyte ratio (NLR) and Monocyte to Lymphocyte ratio (MLR), for diagnosing tuberculosis.

**Methods:**

We retrospectively grouped 114 patients with fever into those diagnosed with TB and control groups. We obtained their haematological data and calculated their derived ratios. The ratios obtained from the two groups were compared. Their sensitivity and specificity were calculated.

**Results:**

Haematological parameters like MLR were higher in TB patients than in the control group. Although NLR was not significantly increased, MLR was significantly increased with p values <0.05. These tests had low sensitivity but high specificity.

**Conclusion:**

Serum NLR and MLR emerge as valuable tools in TB diagnosis. Their simplicity and cost-effectiveness render them particularly suitable for screening and recurrence monitoring in rural and remote settings, thereby mitigating loss to follow-up.

## Introduction

Tuberculosis (TB) is a chronic disease caused by the bacterium Mycobacterium tuberculosis, which has led to significant mortality and morbidity worldwide. Global statistics indicate that 10 million people have been diagnosed with TB. ^1^ In particular, India bears the highest burden of TB, with an estimated 26.4 lakh cases. ^2^ The impact of socioeconomic factors on TB outcomes in India has been well-documented. ^3^

The most common, rapid, and cost-effective diagnostic method for TB in India is the microscopic detection of Acid-Fast Bacilli (AFB) using Ziehl-Neelsen (ZN) staining. However, this method’s sensitivity decreases when the bacterial load is less than 10,000 bacilli/ml, requiring high-quality and large samples. ^4^ AFB culture and nucleic acid amplification tests (NAAT), which are more sensitive, are typically conducted in tertiary centers but are limited in rural areas of developing countries due to resource constraints. ^5^ Moreover, in patients who only produce a small amount of sputum or no sputum at all or in cases of extrapulmonary TB, more interventional methods may be required to obtain adequate samples. Additionally, collection of samples must be done cautiously to avoid contamination, thus demanding technical expertise. While the National Health Mission guidelines suggest diagnosing and treating TB based on high clinical suspicion, studies indicate lower survival rates among patients who receive empirical TB treatment compared to those with laboratory-confirmed TB infection. ^4^ Despite being curable, late diagnosis, ineffective treatment, and loss of follow-up can result in relapse or the development of multidrug-resistant tuberculosis (MDR-TB) or extensively drug-resistant tuberculosis (XDR-TB). Late detection is primarily due to the unavailability of cost-effective and rapid tests in rural areas, exacerbating the TB burden in India. Loss of follow-up and relapse are often attributed to difficulties in accessing these tests. Timely, accurate, and affordable diagnostics are crucially needed, as delays in diagnosis can worsen outcomes and increase transmission rates.

TB is characterized by granulomatous inflammation and caseous necrosis, with neutrophils playing a significant role in its pathogenesis. ^6^^–^^9^ Activated macrophages release tumor necrosis factor (TNF) and chemokines, recruiting more monocytes and ultimately leading to the death of the mycobacterium. ^10^ Hematological parameters such as neutrophils, lymphocytes, and monocytes exhibit variations in TB patients. ^11^^–^^13^ In recent years, ratios derived from various hematological parameters have been explored as markers of inflammation in multiple diseases. NLR and MLR have demonstrated both diagnostic and prognostic value in TB. ^6^^–^^17^ These tests are simple, rapid, cost-effective, and readily available even in rural areas, promising potential as adjunctive biomarkers for diagnosing and screening clinically suspected TB patients and detecting relapse in patients under follow-up.

## Methods

This is an analytical observational case-control study. The study was conducted at our tertiary hospital in Karnataka state of South India, from June to August 2022. The study included inpatients and outpatients who presented with a fever, had undergone laboratory tests in our institutional hospital to rule out both pulmonary and extrapulmonary tuberculosis, and had adequate clinical data including the presence or absence of history of cough, weight loss etc and radiological findings. All pediatric patients below 18 years, terminally ill patients, patients with known comorbidities like diabetes, sero-positive status and those who were on any treatment were excluded from the study. The sampling process involved two stages. At first, a non-probability, convenience sampling method was employed to identify potential participants based on their presentation with fever and undergoing TB testing. Subsequently, purposive sampling was applied to select participants, strictly meeting the inclusion and exclusion criteria to minimize selection bias and ensure the representation of relevant clinical characteristics within each group. In this study, an effect size of 0.5 was chosen based on prior knowledge or expected differences in the variables under investigation, and a power of 80% (or 0.80) was selected. Kelsey’s formula was employed to calculate the sample size required for each group. This formula incorporates the effect size, power, and significance level to determine the minimum sample size needed to detect a significant difference between groups. A minimum sample size of 55 participants per group was calculated and deemed sufficient to detect the specified effect size with the chosen power level. The patients were divided into a TB group if the patients had a fever and tested positive for TB either by AFB culture, ZN stain, or NAAT and a control group if patients had a fever but tested negative for TB by the above-mentioned methods or by radiology or were positive for other infections by culture. The clinical details were procured from the case files. For the hematologic evaluation, for all groups, the following blood parameters: differential neutrophil, lymphocyte, and monocyte counts, were measured by an automated haematology analyser. The NLR was calculated by dividing the differential neutrophil count by the differential lymphocyte count, and the MLR by dividing the differential monocyte count by the differential lymphocyte count. Data was collected from laboratory software or case files and presented as mean±standard deviation (SD). It was recorded using Microsoft Excel. Statistical Package for the Social Sciences, version 29 software was used for statistical analysis. The T-test was used for continuous variables, and the chi-square test for categorical variables. A p-value of <0.05 was considered statistically significant.

### Ethics and consent

We obtained approval from the Institutional Ethics Committee. (Kasturba Medical College, Mangalore), Reg. No. ECR/541/Inst/KA/2014/RR-20, DHR Reg. No. EC/NEW/INST/2020/742 with Protocol No. IECKMCMLR-11/2021/343. The approval was given on 17 ^th^ November 2021. The committee permitted a waiver of consent to participate from the patients as patient details and data were sourced from case files, and tracing the patients proved challenging.

## Results

In total, 114 cases were included in the study, evenly distributed between the TB group (n=57) and the control group (n=57). Key hematologic markers assessed included Neutrophils, Lymphocytes, and Monocytes, with Neutrophil to Lymphocyte Ratio (NLR) and Monocyte to Lymphocyte Ratio (MLR) derived for each participant. ^18^

**Figure 1.**
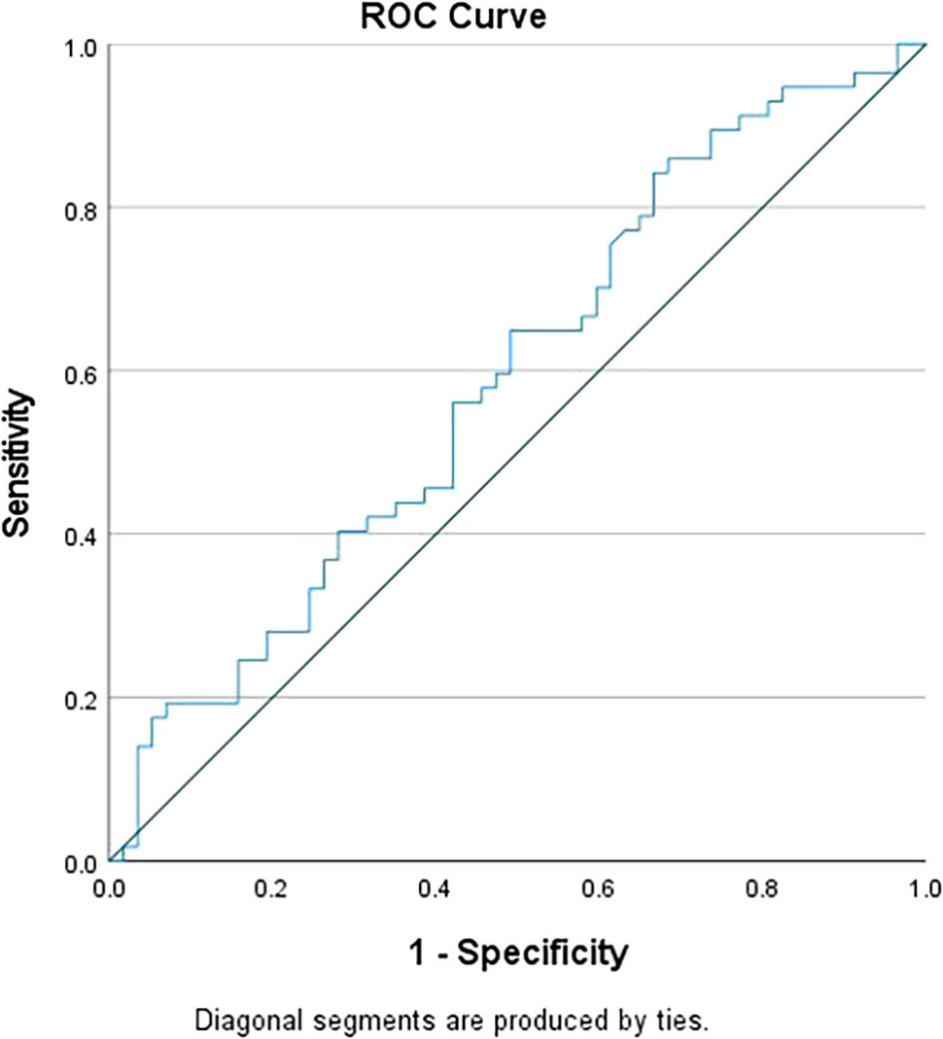
ROC curve for NLR.

**Figure 2.**
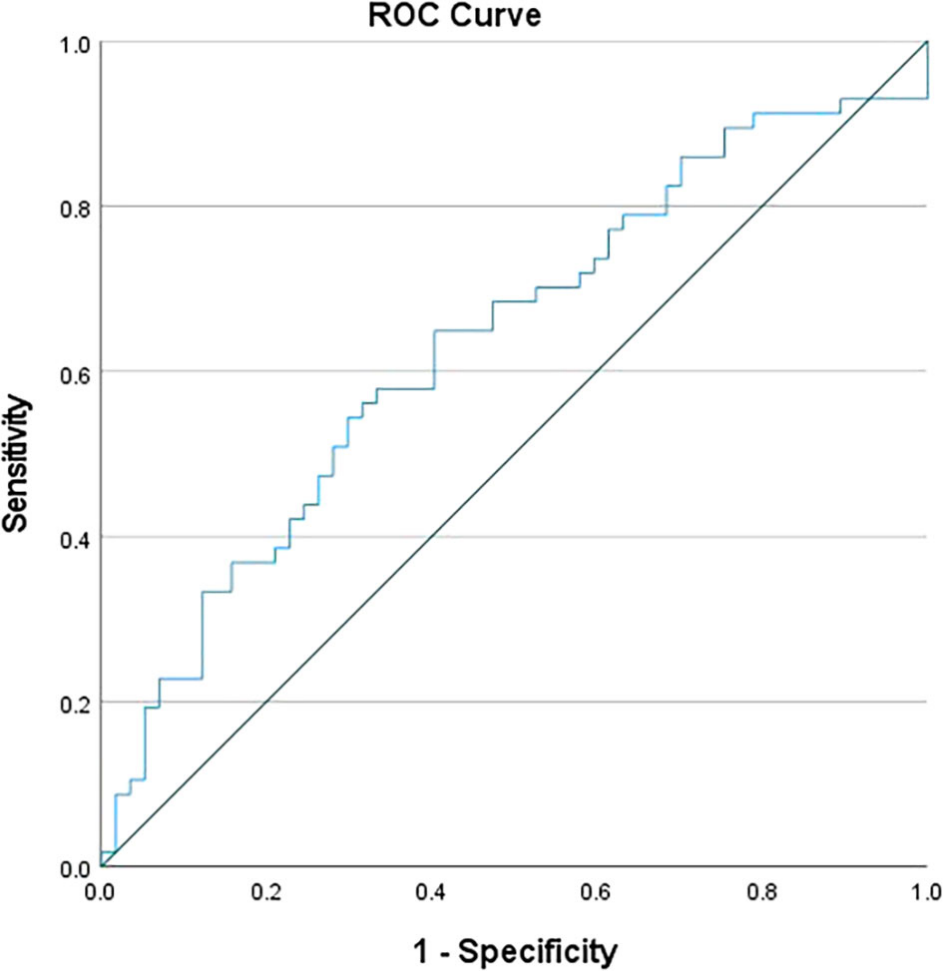
ROC curve for MLR.

No significant differences were observed in any haematological parameters between different age groups or between the two sexes in our study. Differential lymphocyte counts were found to be significantly elevated in TB cases compared to controls (P value=0.048), while MLR showed a significant increase in TB cases compared to controls (P value=0.031). However, no significant differences were observed in the counts of Neutrophils, Monocytes, and NLR between TB cases and controls. To distinguish between TB patients and controls, the cut-off values were established using Receiver Operating Characteristic (ROC) curve analysis. The cut-off value for NLR was set at >5 ( Figure 1), while for MLR, it was set at >0.5 ( Figure 2). Individually, NLR and MLR exhibited limited sensitivity in predicting TB, but demonstrated better specificity. However, when combining these parameters, there was significant increase in specificity, as shown in Table 1. ^19^

**Table 1.** Performance metrics of NLR, MLR, and combined NLR & MLR for predicting tuberculosis.

Metrics	NLR	MLR	NLR & MLR
Sensitivity %	28.0	38.6	22.8
Specificity %	77.2	78.9	96.5
Positive Predictive Value %	55.2	64.7	86.7
Negative Predictive Value %	51.8	56.3	55.6

## Discussion

Tuberculosis remains a significant cause of morbidity worldwide, especially in low-income countries, emphasizing the critical need for accessible and cost-effective diagnostic methods. Despite the availability of rapid tests like the NAAT assay, challenges persist, particularly for diagnosing extra-pulmonary, pediatric, and smear-negative TB cases. Conventional methods such as smear tests are outdated, while serological tests lack accuracy. ^16^^,^^17^

Our study focused on key hematological parameters including neutrophil count, lymphocyte count, monocyte count, neutrophil-to-lymphocyte ratio (NLR), and monocyte-to-lymphocyte ratio (MLR) in patients presenting with fever, comparing those diagnosed with TB to others. Previous research has underscored NLR and MLR as significant markers for TB diagnosis and prognosis. ^6^^–^^16^

In our investigation, MLR emerged as a standout parameter, exhibiting a statistically significant increase in TB patients compared to those without TB. This finding underscores the involvement of activated macrophages and monocytes in chronic granulomatous inflammation characteristic of TB. However, contrary to some prior studies, NLR did not demonstrate a significant role in TB diagnosis, possibly due to the involvement of neutrophils in non-granulomatous and acute inflammatory conditions. While NLR and MLR have shown associations with TB disease severity and treatment outcomes in some studies, their predictive value is not universally consistent. ^14^^–^^17^ Factors like comorbidities, immune status, and TB strain virulence can influence the relationship between these ratios and clinical outcomes. NLR and MLR can also vary significantly between individuals based on factors like age, sex, and ethnicity and can fluctuate over time due to stress, medications, and physiological changes. ^15^

While individual hematological parameters showed limited sensitivity, combining MLR and NLR substantially improved sensitivity, specificity, and positive predictive value. This suggests the potential for a combined approach to aid in TB detection.

Acknowledged study limitations include, unknown hidden comorbidities, unsuspected infections or immune status, unknown stress factors in both control and cases, and the unknown virulence of the TB strain, potentially introducing variability in laboratory findings. Other limitations include the possibility of selection bias in the patient selection process despite strict adherence to inclusion and exclusion criteria due to a lack of relevant investigations of the desired population. Notably, individuals with TB who did not present with fever were excluded, potentially limiting the study’s representativeness of the TB population.

Despite these limitations, our study highlights the importance of utilizing hematological parameters, particularly MLR, in TB diagnosis. While these ratios are non-specific, have inter-individual variation and limited predictive value, limiting their reliability as standalone markers of TB severity or treatment response, combining these ratios with established tests like elevated Erythrocyte Sedimentation Rate (ESR) offers promise for TB screening in resource-limited settings.

## Conclusion

In conclusion, MLR emerges as a promising, cost-effective tool for early TB diagnosis. Utilizing a combined approach with NLR enhances screening efficacy, especially in cases with strong clinical suspicion. Moreover, this combined strategy holds the potential for monitoring patients during follow-up, mitigating relapse risks, and alleviating the TB burden, particularly in underserved regions and rural areas of low-income countries. Variations in study populations, laboratory methods, and analytical techniques contribute to inconsistencies in cutoff thresholds, hindering comparability and generalizability across studies. Therefore this avenue warrants further research to know its hold in clinical implications and also to validate their diagnostic accuracy, prognostic value, and predictive capabilities across diverse patient populations and clinical settings.

## Data Availability

This project contains the following underlying data:
1. Data Excel sheet for Deciphering the significance of neutrophil to lymphocyte and monocyte to lymphocyte ratios in tuberculosis: A case-control study from southern India, Figshare: https://doi.org/10.6084/m9.figshare.23684778.v5 ^18^
2. Statistical analysis of Deciphering the significance of neutrophil to lymphocyte and monocyte to lymphocyte ratios in tuberculosis: A case-control study from southern India, Figshare: https://doi.org/10.6084/m9.figshare.23684859.v5 ^19^ Data are available under the terms of the Creative Commons Zero “No rights reserved” data waiver (CC0 1.0 Public domain

## References

[1] World Health Organization: Tuberculosis (TB).[Accessed 9 February 2022]. Reference Source

[2] TBFacts: TB Statistics, India.[Accessed 9 February 2022]. Reference Source

[3] Bhargava A : *Social determinants of tuberculosis: context, framework, and the way forward to ending TB in India.* Taylor & Francis; [Accessed 26 July 2022]. 10.1080/17476348.2021.1832469?journalCode=ierx20 33016808

[4] Molicotti P Bua A Zanetti S : Cost-effectiveness in the diagnosis of tuberculosis: choices in developing countries. *J. Infect. Dev. Ctries.* 2014;8(01):024–038. 10.3855/jidc.3295 24423709

[5] Singh P : Diagnosis of TB from conventional to modern molecular protocols. *Front. Biosci.* 2019;11(1):38–60. 10.2741/e845 30468637

[6] Yin Y Kuai S Liu J : Pretreatment neutrophil-to-lymphocyte ratio in peripheral blood was associated with pulmonary tuberculosis retreatment. *Arch. Med. Sci.* 2017;13(2):404–411. 10.5114/aoms.2016.60822 28261295 PMC5332451

[7] Han Y Kim SJ Lee SH : High blood neutrophil-lymphocyte ratio associated with poor outcomes in miliary tuberculosis. *J. Thorac. Dis.* 2018 Jan;10(1):339–346. 10.21037/jtd.2017.12.65 29600065 PMC5863179

[8] Miyahara R Piyaworawong S Naranbhai V : Predicting the risk of pulmonary tuberculosis based on the neutrophil-to-lymphocyte ratio at TB screening in HIV-infected individuals. *BMC Infect. Dis.* 2019;19:667. 10.1186/s12879-019-4292-9 31357936 PMC6664723

[9] Iliaz S Iliaz R Ortakoylu G : Value of neutrophil/lymphocyte ratio in the differential diagnosis of sarcoidosis and tuberculosis. *Ann. Thorac. Med.* 2014 Oct;9(4):232–235. 10.4103/1817-1737.140135 25276243 PMC4166071

[10] Chen G Wu C Luo Z : Platelet-lymphocyte ratios: a potential marker for pulmonary tuberculosis diagnosis in COPD patients. *Int. J. Chron. Obstruct. Pulmon. Dis.* 2016 Nov 3;11:2737–2740. 10.2147/COPD.S111254 27843310 PMC5098523

[11] Iqbal S Ahmed U Khan MA : Haematological parameters altered in tuberculosis. *Pak. J. Physiol.* 2015 Mar 31;11(1):13–16.

[12] Okeke CO Amilo GI Ifeanyichukwu MO : Longitudinal assessment of the impact of tuberculosis infection and treatment on monocyte–lymphocyte ratio, neutrophil-lymphocyte ratio, and other white blood cell parameters. *Egypt. J. Haematol.* 2020 Apr 1;45(2):97. 10.4103/ejh.ejh_62_19

[13] Yoon NB Son C Um SJ : Role of the neutrophil-lymphocyte count ratio in the differential diagnosis between pulmonary tuberculosis and bacterial community-acquired pneumonia. *Ann. Lab. Med.* 2013 Mar 1;33(2):105–110. 10.3343/alm.2013.33.2.105 23482854 PMC3589634

[14] Jeon Y Lee WI Kang SY : Neutrophil-to-Monocyte-Plus-Lymphocyte Ratio as a Potential Marker for Discriminating Pulmonary Tuberculosis from Nontuberculosis Infectious Lung Diseases. *Lab. Med.* 2019 Jul 16;50(3):286–291. 10.1093/labmed/lmy083 30753566

[15] Rees CA Pineros DB Amour M : The potential of CBC-derived ratios (monocyte-to-lymphocyte, neutrophil-to-lymphocyte, and platelet-to-lymphocyte) to predict or diagnose incident TB infection in Tanzanian adolescents. *BMC Infect. Dis.* 2020;20(1):609. 10.1186/s12879-020-05331-w 32811463 PMC7433160

[16] The Lancet Infectious Diseases. *Correction to Lancet Infect. Dis.* 2020. published online Feb 18. 10.1016/S1473-3099(20)30128-6 PMC7128727 32105640

[17] Jarosławski S Pai M : Why are inaccurate tuberculosis serological tests widely used in the Indian private healthcare sector? A root-cause analysis. *J. Epidemiol. Glob. Health.* 2012;2(1):39–50. 10.1016/j.jegh.2011.12.001 23856397 PMC7320362

[18] Nayak R Rai S : Data Excel sheet for Deciphering the significance of neutrophil to lymphocyte and monocyte to lymphocyte ratios in tuberculosis: A case-control study from southern India. figshare.[Dataset].2024. 10.6084/m9.figshare.23684778.v5 PMC11786103 39895948

[19] Nayak R Rai S : Statistical analysis for Deciphering the significance of neutrophil to lymphocyte and monocyte to lymphocyte ratios in tuberculosis: A case-control study from southern India. figshare.[Dataset].2024. 10.6084/m9.figshare.23684859.v5 PMC11786103 39895948

