# Supplementary material for: Deciphering the significance of neutrophil to lymphocyte and monocyte to lymphocyte ratios in tuberculosis: A case-control study from southern India
Source: F1000Res. 2025 Jan 8;13:747. Originally published 2024 Jul 5. [Version 2] doi: 10.12688/f1000research.150685.2 (PMC11786103; doi:10.12688/f1000research.150685.2)
Supplement: Peer review history

# Reviewer report: Reviewer response for version 2

**DOI:** 10.5256/f1000research.176494.r357265

**Published:** 2025-01-31

**Authors:** Tutik Tutik Kusmiati

**Affiliations:** 1 Universitas Airlangga – Dr. Soetomo General Academic Hospital, Surabaya, Indonesia

The manuscript has been appropriately updated. I have no further comment.

Is the work clearly and accurately presented and does it cite the current literature?

Yes

If applicable, is the statistical analysis and its interpretation appropriate?

Partly

Are all the source data underlying the results available to ensure full reproducibility?

Partly

Is the study design appropriate and is the work technically sound?

Yes

Are the conclusions drawn adequately supported by the results?

Partly

Are sufficient details of methods and analysis provided to allow replication by others?

Partly

Reviewer Expertise:

Pulmonology and Respiratory Medicine

I confirm that I have read this submission and believe that I have an appropriate level of expertise to confirm that it is of an acceptable scientific standard.

# Reviewer report: Reviewer response for version 2

**DOI:** 10.5256/f1000research.176494.r357264

**Published:** 2025-01-17

**Authors:** Sivaporn Gatechompol

**Affiliations:** 1 HIV-NAT, Thai Red Cross AIDS Research Centre, Bangkok, Thailand

The manuscript has been appropriately updated.

 I have no further comment.

Is the work clearly and accurately presented and does it cite the current literature?

Partly

If applicable, is the statistical analysis and its interpretation appropriate?

Partly

Are all the source data underlying the results available to ensure full reproducibility?

Partly

Is the study design appropriate and is the work technically sound?

Partly

Are the conclusions drawn adequately supported by the results?

Partly

Are sufficient details of methods and analysis provided to allow replication by others?

No

Reviewer Expertise:

Infectious disease, focus on TB and HIV

I confirm that I have read this submission and believe that I have an appropriate level of expertise to confirm that it is of an acceptable scientific standard.

# Response

**Published:** 2025-01-17

**Authors:** Rakshatha Nayak

**Affiliations:** Kasturba Medical College Hospital, India

Dear Madam, 

 Thank you for reviewing our article.

# Reviewer report: Reviewer response for version 1

**DOI:** 10.5256/f1000research.165277.r336227

**Published:** 2024-11-13

**Authors:** Tutik Tutik Kusmiati

**Affiliations:** 1 Universitas Airlangga – Dr. Soetomo General Academic Hospital, Surabaya, Indonesia

in abstract: I agree that ZN has low sensitivity, but the culture sensitivity is very good.

 in introduction: In the introduction: you only wrote the reason for studying MLR because ZN is not sensitive and the source of funds for molecular examination is expensive. In my opinion, a more appropriate reason is: the difficulty of obtaining sputum samples to evaluate treatment because in more than 2 weeks of treatment the patient's complaints have decreased so that it is difficult to produce sputum because they are no longer coughing

 in Method: In the control group that was included only fever, why was the cough not asked, why was a chest X-ray not performed to rule out that the patient really did not have TB?

 As we know, there is clinically confirmed TB, namely if there are complaints that point to TB, the CXR results support TB but the bacteriological results are negative. 

 To minimize bias, do you exclude patients with Diabetes and HIV because they are immunocompromised patients which can affect MMR and NLR values?

Is the work clearly and accurately presented and does it cite the current literature?

Yes

If applicable, is the statistical analysis and its interpretation appropriate?

Partly

Are all the source data underlying the results available to ensure full reproducibility?

Partly

Is the study design appropriate and is the work technically sound?

Yes

Are the conclusions drawn adequately supported by the results?

Partly

Are sufficient details of methods and analysis provided to allow replication by others?

Partly

Reviewer Expertise:

Pulmonology and Respiratory Medicine

I confirm that I have read this submission and believe that I have an appropriate level of expertise to confirm that it is of an acceptable scientific standard, however I have significant reservations, as outlined above.

# Response

**Published:** 2024-12-30

**Authors:** Rakshatha Nayak

**Affiliations:** Kasturba Medical College Hospital, India

Dear Madam,

 Thank you for reviewing our article and providing valuable feedback. In response to your suggestions, we have made the following revisions:

1. We have revised the abstract to mention the other disadvantages of AFB culture, as opposed to focusing solely on its sensitivity, as per your recommendation.
2. We agree with your point that obtaining sputum can be difficult in patients who do not present with a cough. We have added this concern as an additional point in the introduction to highlight the challenges involved.
3. The study involved all patients presenting with fever, including those with extrapulmonary TB. Therefore, while we did inquire about symptoms such as cough and other clinical features, these were not specifically emphasized in the article. Following your suggestion, we have now included this information under the inclusion criteria in the methodology section (Version 2).
4. We selected controls based on clinical, radiological, microbiological, and molecular findings to ensure that only true negative TB cases were included. We did not include TB patients without fever, as the subjects were selected from a fever OPD, which is mentioned in the study's limitations under discussion. Despite this limitation, we believe the study remains highly relevant, given that fever is the most common symptom in both pulmonary and extrapulmonary TB cases.
5. We have excluded patients with known comorbidities as mentioned in Version 1. But as per your suggestion, we have specified few such as diabetes and HIV, as noted in the exclusion criteria section of the article in version 2.
6. The article has been revised in accordance with the suggestions provided by the reviewers. We kindly request you to review the updated version of the article and share your valuable comments and approval. We have put sincere efforts into refining the study to enhance its accuracy and applicability, particularly in remote areas, to help reduce the global burden of tuberculosis.

We look forward to your feedback.

# Reviewer report: Reviewer response for version 1

**DOI:** 10.5256/f1000research.165277.r308652

**Published:** 2024-08-21

**Authors:** Sivaporn Gatechompol

**Affiliations:** 1 HIV-NAT, Thai Red Cross AIDS Research Centre, Bangkok, Thailand

I am pleased to review this work. This is a case-control study in a tertiary hospital in India. The study aimed to assess the usefulness of  Neutrophil to Lymphocyte ratio (NLR) and Monocyte to Lymphocyte ratio (MLR) for diagnosing tuberculosis.

 I have some comments as following

 Methods

 1. The TB case definition is not clear. Which type of TB were included in the study (i.e pulmonary, extrapulmonry, or both) ? 

 2. What is the HIV status of the participants ? Since HIV has direclty effect on  both NLR and MLR.  

 3. The patients who had fever but tested negative for TB by AFB culture, ZN stain, or NAAT were assigned to the control group. How can the authors exclude smear/culture negative TB for this group ? 

  Results 

 1. The authors mentioned in discussion that NLR and MLR can also vary significantly between individuals based on factors like age, sex, and ethnicity. But the authors did not provide these informations in the results or compare between cases and controls.  

Is the work clearly and accurately presented and does it cite the current literature?

Partly

If applicable, is the statistical analysis and its interpretation appropriate?

Partly

Are all the source data underlying the results available to ensure full reproducibility?

Partly

Is the study design appropriate and is the work technically sound?

Partly

Are the conclusions drawn adequately supported by the results?

Partly

Are sufficient details of methods and analysis provided to allow replication by others?

No

Reviewer Expertise:

Infectious disease, focus on TB and HIV

I confirm that I have read this submission and believe that I have an appropriate level of expertise to state that I do not consider it to be of an acceptable scientific standard, for reasons outlined above.

# Response

**Published:** 2024-12-30

**Authors:** Rakshatha Nayak

**Affiliations:** Kasturba Medical College Hospital, India

Dear Madam,

 Thank you for reviewing our article and providing us with your valuable insights. In response to your suggestions, we have made the following revisions:

1. The study included all patients with fever, encompassing both pulmonary and extrapulmonary TB cases. This information has been added to the inclusion criteria in the methodology section.
2. We have excluded patients with known comorbidities as mentioned in Version 1. But as per your suggestion, we have specified few such as diabetes and HIV, as noted in the exclusion criteria section of the article in version 2
3. The controls were classified as true negatives after evaluating all clinical, radiological, microbiological, and molecular findings. This update has been reflected in the methodology section.
4. There was no significant difference between age groups or sexes, and we have updated the results accordingly. Regarding ethnicity, we acknowledge the complexity of making comparisons in India due to the immense diversity across ethnic, linguistic, religious, and cultural lines, as well as historical, social, political, and genetic factors. As such, broad comparisons or generalizations about ethnicity would oversimplify the situation, and we have refrained from including such comparisons in the article.
5. The article has been updated as per the suggestions provided by the reviewers. We kindly request you to review the updated version of the article and share your valuable feedback and approval. We have put sincere efforts into refining the study to enhance its accuracy and applicability, particularly in remote areas, to help reduce the global burden of tuberculosis.

We look forward to your feedback.
